# The Human Alpha3 Beta2 Neuronal Nicotinic Acetylcholine Receptor Can Form Two Distinguishable Subtypes

**DOI:** 10.3390/ijms26199506

**Published:** 2025-09-28

**Authors:** Doris C. Jackson, Marcel K. Hall, Sterling N. Sudweeks

**Affiliations:** Department of Cell Biology and Physiology, Brigham Young University, Provo, UT 84602, USA; dorisgclark@gmail.com (D.C.J.); marcelkhall@gmail.com (M.K.H.)

**Keywords:** nicotinic, acetylcholine, hippocampus

## Abstract

Diverse neuronal nicotinic acetylcholine receptor (nAChR) subtypes are expressed in hippocampal interneurons. Single-cell analysis of mRNA expression previously revealed prominent co-expression of the α3 and β2 subunits within rat interneurons in the CA1 region. Although the α3 subunit (traditionally expressed together with β4) is usually associated with the peripheral nervous system, its significant co-expression with the β2 subunit in hippocampal interneurons suggests a distinct, potentially novel central nervous system nAChR subtype. We demonstrate that the human α3 and β2 subunits injected into *Xenopus laevis* oocytes can assemble into at least two functionally distinct subtypes of nAChRs based on different subunit stoichiometries. These subtypes exhibit similar reversal potentials but differ significantly in their desensitization kinetics and acetylcholine (ACh) affinities. The response obtained from a 1:5 α3:β2 mRNA injection ratio shows a higher affinity for ACh and significantly greater desensitization during prolonged ACh application compared to the response obtained from a 5:1 α3:β2 mRNA injection ratio. The identification of distinct functional α3β2 subtypes, characterized by differential desensitization kinetics and ACh affinity, could represent novel targets for the potential development of highly selective cognitive therapeutics for conditions such as Alzheimer’s disease, autism spectrum disorder, and attention deficit hyperactivity disorder, where hippocampal nAChRs are implicated.

## 1. Introduction

Neuronal nicotinic acetylcholine receptors (nAChRs) comprise a family of related genes (α2–α10, β2–β4) that can assemble to form pentameric ion channels that act as neurotransmitter receptors. Among their functions in the brain, neuronal nAChRs found in the interneurons of the hippocampus [1,2,3] play an important role in regulating the synchronous firing of the much more numerous hippocampal pyramidal cells [4,5]. The nAChR population in hippocampal interneurons is extremely heterogeneous and interneurons can have a diverse array of nAChR subunit mRNA expression [6,7]. Patch clamp recordings from interneurons in the rat hippocampus also reveal a range of electrophysiological properties, indicating a large diversity of receptors subtypes [6]. Interestingly, among all that diversity, single-cell analysis of nAChR subunit mRNA co-expression in the rat hippocampus indicates that the α3 and β2 subunits are the most commonly co-expressed subunits within the stratum oriens and stratum radiatum of the CA1 hippocampus [6], indicating that these subunits might contribute to one of the major receptor subtypes expressed by these neurons. The α3 subunit is traditionally thought of as mainly a peripheral nervous system (PNS) nicotinic subunit, where it has high levels of co-expression with the β4 subunit in the autonomic ganglia of both the sympathetic and parasympathetic pathways in the PNS. The α3 subunit shows very limited expression in the central nervous system (CNS), with the majority of its expression appearing in the medial habenula [8]. In both the PNS and the CNS, the α3 subunit is usually co-expressed with the β4 subunit to make functional channels (reviewed in [8,9,10]). Many neuronal nAChR subunits have shown the ability to produce multiple functional subtypes of receptors using different subunit stoichiometries; for examples, see [11,12,13,14,15,16,17]. The previously reported single-cell co-expression of α3 with the β2 nAChR subunit mRNAs in hippocampal interneurons [6] could represent a relatively specific hippocampus subtype of nAChR. In this report we demonstrate that there are at least two different functional subtypes of α3 and β2 nAChRs, based on differences in subunit stoichiometry.

Nicotinic agonists have a role in enhancing cognition (see [18,19,20,21] for example review articles). One obvious target for this effect would be nAChRs in the hippocampus, a brain structure that plays a strong role in cognition. Hippocampal interneurons, even though they are much smaller in number compared to the excitatory pyramidal cells and non-neuronal glial cells in the hippocampus, would be the likely target of this pro-cognitive effect, since they express nAChRs [2,6,7]. Cognitive therapeutics targeting nAChRs in the hippocampus are often thought to work through either the α7 containing nAChRs [22,23,24,25] or the α4β2 nAChR [26,27]. Both α7 homomers and α4β2 heteromers are found widely throughout the brain. However, the α4 subunit mRNA was expressed at relatively low levels among the rat CA1 hippocampal interneurons [6]. Due to the high co-expression levels of α3β2 mRNA in rat hippocampal interneurons, this receptor subtype represents a potential new drug target that should be fully investigated.

The α3β2 nAChR has been initially characterized by several groups [28,29,30,31,32,33], but the distinction between possible α3β2 subtypes has not been fully explored. Like the α4β2 and the α2β2 nAChRs [16,33,34,35], we demonstrate that there are at least two functional stoichiometries of the α3β2 nAChR. This suggests that different subtypes of α3β2 nAChRs could potentially serve as therapeutic targets for various diseases affecting the hippocampus, such as Alzheimer’s disease (AD) [19,36,37], autism spectrum disorder (ASD) [36,38], and attention deficit hyperactivity disorder (ADHD) [39,40]. Therefore, we sought to characterize the kinetic properties of two subtypes of α3β2 nAChRs and find ways to differentiate them electrophysiologically.

## 2. Results

**Figure 1:**
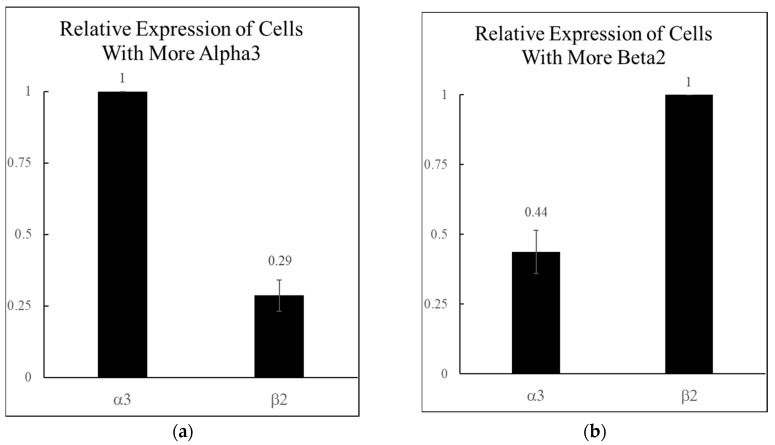
Relative levels of α3 and β2 nicotinic acetylcholine receptor (nAChR) mRNA expression in rat hippocampal interneurons. The most commonly co-expressed α and β subunits from Jackson et al. 2024 [6] were the α3 and β2 subunits (n = 36 neurons). These were analyzed further to identify two populations, depending on which subunit was expressed higher. (**a**) A total of 56% of the α3β2 expressing neurons had more α3, with a 3.45-fold α3:β2 mRNA ratio (n = 20 for (**a**)). (**b**) A total of 44% of the α3β2 expressing neurons had more β2 with a 2.27-fold α3:β2 mRNA ratio (n = 16 for (**b**)).

Previously reported single-cell RT-qPCR data indicated that the α3 and β2 nAChR subunits are the highest co-expressed α and β subunits in the rat CA1 hippocampal interneurons found in the stratum oriens and stratum radiatum [6]. Further examination of this data showed that 56% of the neurons co-expressing α3 and β2 had a much greater α3 expression (3.45-fold more), while the other 44% had a much greater β2 expression (2.27-fold more). Therefore, the two populations showed either a roughly 3:1 or a 1:4 ratio of α3:β2 mRNA on average (Figure 1).

**Figure 2:**
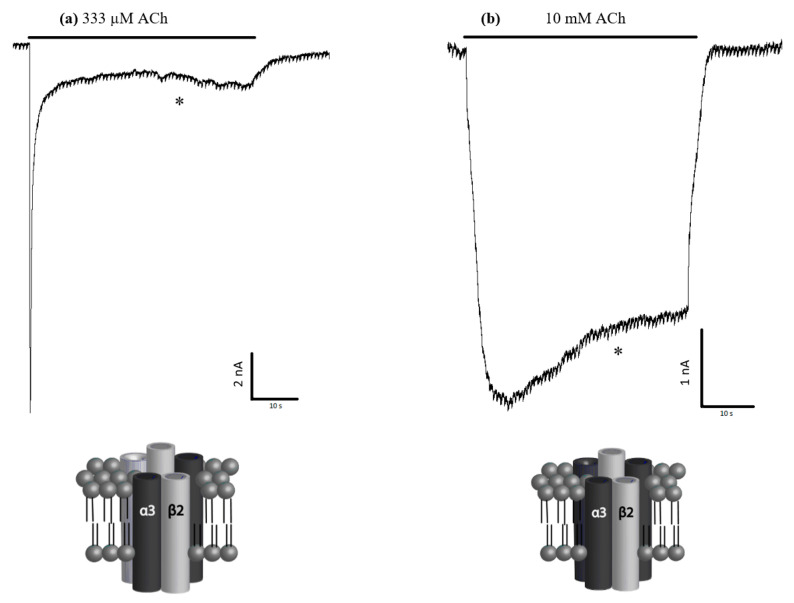
Sample traces and likely stoichiometries. Injection of hα3 and hβ2 nicotinic acetylcholine receptor (nAChR) mRNA into *Xenopus laevis* oocytes at ratios of (**a**) 1:5 and (**b**) 5:1 formed functional and kinetically distinguishable receptors. The respective oocytes were perfused with 333 µM and 10 mM acetylcholine (ACh) (EC_85_) for up to 60 s to characterize desensitization. The percentage of the peak currents remaining after 30 s of sustained ACh application (30 s mark indicated by *) were compared and showed a significant difference between the two injected α3β2 ratios (see also Figure 1). Graphical representations of the likely potential stoichiometries are shown below each respective electrophysiological trace (i.e., either α3_(2)_ + β2_(3)_ or α3_(3)_ + β2_(2)_), modeled on what is already published about stoichiometries for α4β2 and the α2β2 nAChRs [16,33,34,35].

Injection of hα3 and hβ2 mRNA into *Xenopus laevis* oocytes at a 1:5 and 5:1 α3:β2 (the 5× ratios were used to help force differences in receptor stoichiometry) revealed two functional receptor subtypes. Both subtypes responded to ACh application. Extended ACh applications (30 s) at their respective EC_85_ revealed distinct kinetic differences in receptor desensitization (Figure 2). The 5:1 α3:β2-injected oocytes showed remarkably less desensitization to extended applications of ACh. We compared the percentage of the initial peak current still remaining at the 30 s mark between the two subtypes using a *t*-test, which indicated statistical significance (*p* < 0.001). Example recordings showing the marked difference in desensitization to extended applications of ACh are shown in Figure 2.

**Figure 3:**
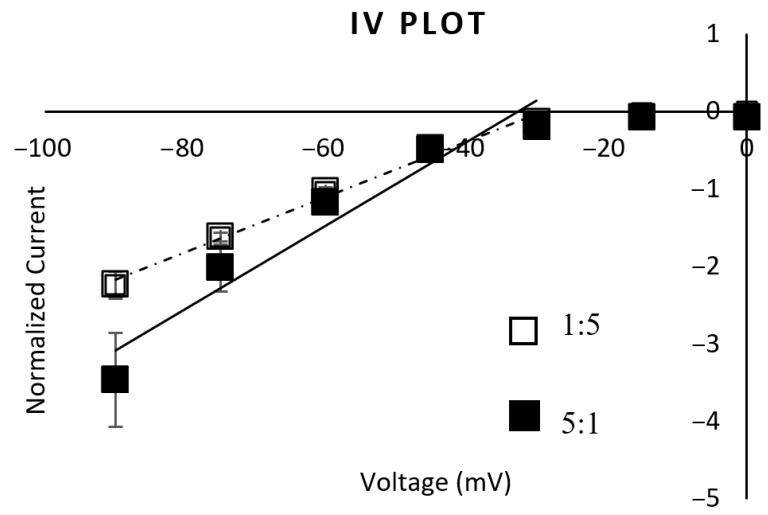
IV plot. The IV plot obtained from the α3:β2 1:5 mRNA-injected oocytes fit a linear trendline of y = 0.0356x + 1.033 (R^2^ = 0.9892) (dashed line, n = 5). The IV plot obtained from the α3:β2 5:1 mRNA-injected oocytes fit a linear trendline of y = 0.0537x + 1.7478 (R^2^ = 0.9362) (solid line, n = 8) when fitting the points between −90 mV and −30 mV (the linear part of the rectifying plot). The reversal potential for both is approximately −30 mV, with a strong inward rectification. ANOVA testing showed no significant difference between these IV responses.

The IV plot shows a similar reversal potential between the subtypes, suggesting that there is no difference in ion selectivity. Also, the reversal potential suggests that, like other nAChR subtypes, the channels are permeable to both Na^+^ and K^+^ (Figure 3). The IV plot also shows that both subtypes are strong inwardly rectifying channels.

**Figure 4:**
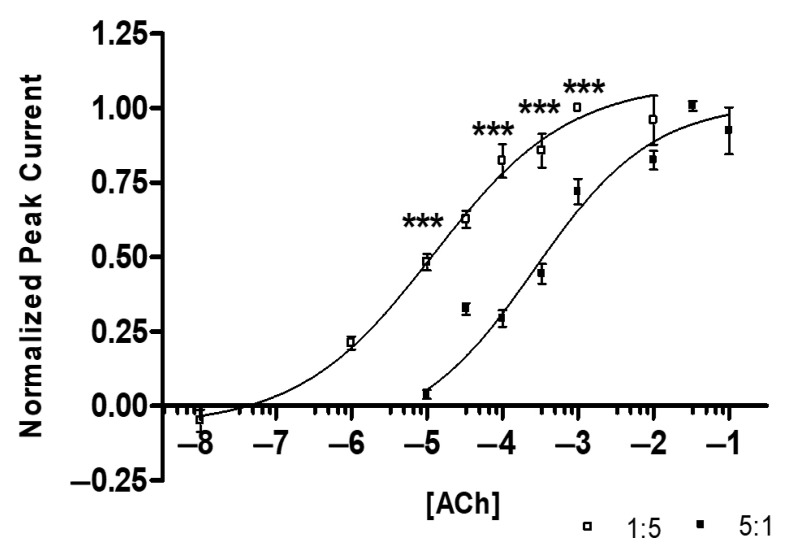
Acetylcholine (ACh) Dose–Response Curves: 1:5 (α3:β2) EC_50_ = 12.2 ± 1.7 μM, n_H_ = 0.49 ± 0.13 (R^2^ = 0.74) (n = 14 oocytes, replicates of 4, 1 individual data point identified as an outlier and removed), 5:1 (α3:β2) EC_50_ = 263.8 ± 1.6 μM, n_H_ = 0.55 ± 0.15 (R^2^ = 0.77) (n = 12 oocytes, replicates of 4, 2 individual data points outliers). Grubb’s outlier tests were used for outliers (alpha = 0.05). The 1:5 injected ratio has a minimum response at ~100 nM and an E_max_ at ~1000 µM ACh. The 5:1 injected ratio has a minimum response at ~10 µM and an E_max_ at ~33 mM ACh (ANOVA, F_[15,258]_ = 54.644, *** *p* < 0.001).

The dose–response curves (Figure 4) reveal a statistical difference in the ACh affinities when comparing subtypes (comparison of EC-50 values with ANOVA yields *p* < 0.0001, Table 1). The 1:5 α3:β2-injected ratio has a significantly greater affinity for ACh than the 5:1 α3:β2-injected group.

In addition, we used *t*-tests to compare the peak amplitude for each injection ratio. When comparing the EC_85_ and the EC_50_ values on the ACh dose–response curve, the peak amplitudes for the 5:1 α3:β2 are statistically different at each (*p* < 0.0001, *p* < 0.001), with the 5:1 α3:β2 injections consistently resulting in larger currents.

**Figure 5:**
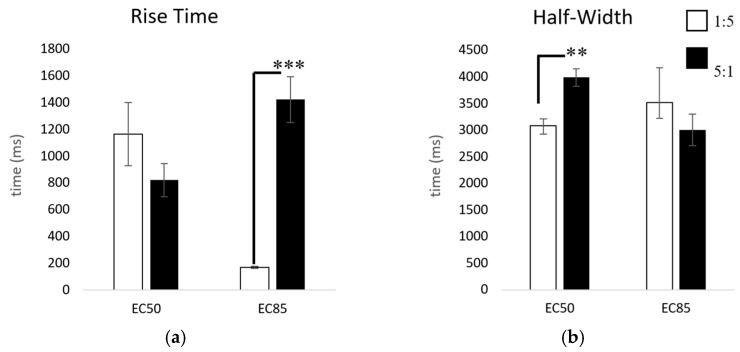
Comparison of rise times and half-widths. Comparisons were made at the EC_50_ (10 µM, 333 µM acetylcholine) and the EC_85_ (333 µM, 10 mM) for the 1:5 and 5:1 α3:β2 mRNA-injected ratios, respectively, using *t*-tests for significance. (**a**) Comparison of 10% to 90% rise time: 1:5 10 µM ($\bar{x}$ = mean ± SEM) $\bar{x}$ = 1166 ± 237 ms (n = 6, replicates of 3), 333 µM $\bar{x}$ = 169 ± 9 ms (n = 21, replicates of 4, 3 replicate outliers removed), 5:1 333 µM $\bar{x}$ = 821 ± 123 ms (n = 13, replicates of 4), 10 mM $\bar{x}$ = 1424 ± 171 ms (n = 25, replicates of 4, 3 replicate outliers removed). (**b**) Comparison of half-width: 1:5 10 µM $\bar{x}$ = 3084 ± 130 ms (n = 5, replicates of 3, 3 replicate outliers removed), 333 µM $\bar{x}$ = 3522 ± 654 ms (n = 11, replicates of 4), 5:1 333 µM $\bar{x}$ = 3988 ± 163 ms (n = 13, replicates of 4, 2 replicate outliers removed), 10 mM $\bar{x}$ = 3003 ± 297 ms (n = 21, replicates of 4) (** *p* < 0.01, *** *p* < 0.001). Grubb’s outlier tests were used to identify significant outliers (alpha = 0.05).

**Figure 6:**
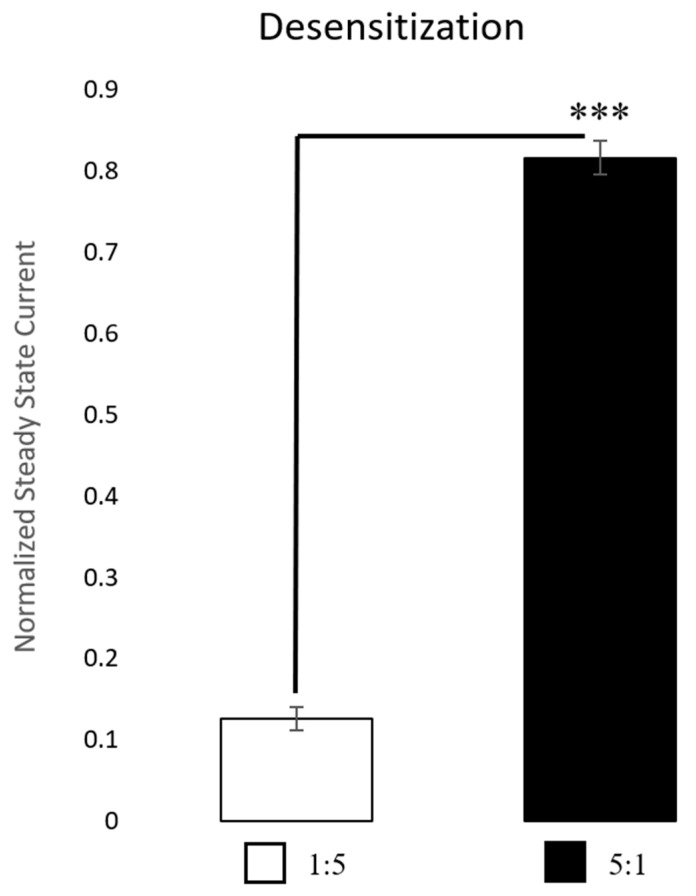
Desensitization. Comparisons were made after 30 s of continuous acetylcholine (ACh) application at the EC_85_ for each receptor subtype. The 1:5 α3:β2-injected oocytes (n = 7 oocytes, performed in replicates of 3 traces, 1 replicate outlier removed) were much more desensitized, withless current remaining compared to the original peak, while the 5:1 α3:β2-injected oocytes (n = 3 oocytes, performed in replicates of 3 traces, 1 replicate outlier removed) were only desensitized minimally with much of the current remaining at 30 s compared to the original peak. Outliers were identified using Grubb’s outlier test (alpha = 0.05). A *t*-test was used to test for the significance of the amount of peak current remaining after 30 s of ACh administration (*** *p* < 0.001).

Our results of the kinetic properties show that when applying ACh for only 3 s, the two subtypes are more similar than different. However, there are at least two parameters that are statistically significant: the half-widths and rise times (Figure 5). Yet, these results can differ depending on ACh concentration. Figure 6 reveals the most distinguishable characteristic between the two populations: with extended ACh applications (i.e., >10 s), the two subtypes show marked differences in desensitization. The ACh responses in 1:5 α3:β2-injected oocytes are significantly more desensitized at their steady state than those in the 5:1 α3:β2-injected oocytes. After 30 s, only 12.6 ± 1.4% of the current compared to the original peak amplitude remained in the 1:5 α3:β2-injected oocytes while 81.6 ± 2.1% ($\bar{x}$ ± SEM) remained in the 5:1 α3:β2-injected oocytes (Figure 6).

## 3. Discussion

The β2 nAChR subunit has been shown to be widely expressed throughout much of the rat brain [41]. However, expression of the α3 subunit is much more restricted, being previously identified mainly in the autonomic ganglia of the PNS with some presence also in the medial habenula of the CNS [8,9,42,43,44,45]. The α3 subunit has also been identified in the modified sympathetic ganglion in the adrenal gland [46], thymus [47], respiratory epithelial cells [48], and keratinocytes [49,50]. There have been some studies performed on α3β2, α3β4, α3α5β2, and α3α5β4 [51,52,53,54]; however, we believe this is the first report in which the α3β2 subunits can form distinct functional receptor subtypes based on different subunit stoichiometries.

The α3 subunit has often been avoided as a pharmacological target, given its high expression in autonomic ganglia of the PNS and potential side effects of modulating those receptors in both the sympathetic and parasympathetic systems at the same time. However, the co-expression of α3 is shown to be much more correlated with the β4 subunit than the β2 in the PNS [8,9,10,53]. Single-cell qPCR data strongly suggests the hippocampal co-expression of the α3 subunit, together with the β2 subunit mRNAs, perhaps also together with the α5 subunit [6]. Additionally, the α5 has a limited expression pattern in both the PNS and CNS. Therefore, the subunit combination of α3α5β2 could also potentially generate unique interfaces (α3β2 and α5β2) that could perhaps be exploited pharmacologically to target hippocampus specific nAChRs.

When considering how other nAChR subtypes assemble together with the single-cell RT-qPCR data from Jackson et al. [6], the data suggest that there are two likely stoichiometries: the α3_(2)_β2_(3)_ and the α3_(3)_β2_(2)_ [14,16,33,34]. The 1:5 and 5:1 mRNA injection ratios will likely not produce a homogenous population of one stoichiometry, but our results indicate that by injecting different subunit ratios, it is possible to force the occurrence of enough of each receptor subtype to distinguish their kinetic properties using whole-cell electrophysiology. It has been shown previously [55,56] that sometimes, a low or broad Hill co-efficient (n_H_) may indicate multiple contributing stoichiometries, but considering the error bars and the fit of our curves, we are probably obtaining very little contribution from the less likely stoichiometries of α3_(1)_β2_(4)_ and α3_(4)_β2_(1)_.

The dose–response curves shown in Figure 4 are likely obtained from a mixture of at least two α3β2 subtypes. Although the best fit for our dose–response curves is monophasic, it is possible that a biphasic fit could be made with another ligand, similar to those found with the α4β2 and α2β2 stoichiometry studies [57,58,59,60]. The 5:1 mRNA injection would increase the likelihood of α3_(3)_β2_(2)_ subtypes expression over the α3_(2)_β2_(3)_ subtype expression. In addition, our results indicating larger overall peak amplitudes for the 5:1injected oocytes may suggest that the α3_(3)_β2_(2)_ is either more efficient in functional receptor protein formation or perhaps it has greater conductance, resulting in overall larger peak amplitudes. Another explanation for the larger peak amplitude size with the 5:1-injected oocytes is that there may be another possible binding site at the α3/α3 junction. Like the α4β2 nAChR, when more α4 is present, an additional binding site is found at the α4/α4 junction [13]. To determine if protein efficiency, conductance, or an additional ACh binding site are contributing to the difference in peak amplitude size, additional experiments would be necessary.

The most noteworthy difference between the two injected subgroups is what is observed for ACh desensitization with prolonged ACh application. The ACh responses from the 1:5-injected oocytes desensitize significantly more than the 5:1-injected oocytes. This desensitization profile is different from the α4β2 nAChR published results. The pattern of desensitization observed with the α4β2 nAChR subtypes shows that more β2 subunit expression decreases desensitization, whereas we found that more β2 subunit expression increased desensitization with α3 [11,14,16]. However, similar to the α4β2 and α2β2 ACh dose response curves, increasing the number of β2 subunits in the receptor increases the ACh affinity and shifts the dose–response curve left [14,16,33,34].

To summarize, the α3β2 appears to be more restricted to hippocampal interneurons than the α7 or the α4β2 subtypes; therefore, drugs targeting the α3β2* (the * here indicates other potential subunits may be included in various pentameric subtypes as well) may be more selective than current therapies. We can now add it to the list of nAChR subtypes that should be screened for cognitive drug development, as was also indicated recently by work undertaken by Ota et al. [61]. Many cognitive diseases like AD and ASD currently have few effective treatment options. Therefore, characterization of a new protein target in the hippocampus may widen the possible therapeutics and give further insight into disease mechanisms. We demonstrate that multiple α3β2 nAChR subtypes exist in the hippocampus, and although similar in many respects, they do have distinguishable properties. Both subtypes should be considered valid novel targets for future cognitive therapies.

## 4. Materials and Methods

Plasmids containing human α3 (hα3) and human β2 (hβ2) genes were transformed into One Shot^®^ *E. coli* chemically competent cells, and plasmid DNA was then isolated and purified using the HiSpeed^®^ plasmid purification kit. Plasmids containing the hα3 and hβ2 genes were linearized by restriction digest with SacI. The mRNA was then transcribed, capped on the 5′ end, a poly(A) tail was added, and LiCL purification was performed using the mMessage mMachine^®^ T7 Ultra Kit according to the protocol provided. RNA was re-suspended in Tris-EDTA Buffer, mixed in various ratios, aliquoted, and stored at −20 °C.

Ratios of hα3 (1.0 µg/µL) and hβ2 (1.0 µg/µL) nAChR subunit mRNAs were injected into *Xenopus laevis* oocytes. Naive *Xenopus laevis* oocytes are not responsive to ACh, but when injected with the appropriate mRNA, they are also able to express functional nAChRs. The ratios of α3:β2 1:5 and α3:β2 5:1 were used to increase the likelihood of expression of each probable stoichiometry (α3_(2)_β2_(3)_ and α3_(3)_β2_(2)_, respectively), similar to what has been achieved with the α4β2 nAChR [13,14,62]. Each oocyte was injected with 50.6 nL of mRNA for a total of 50.6 ng of mRNA per oocyte using a Nanoject II (Drummond Scientific Company). The injection needles and recording electrodes were made of borosilicate capillary glass tubes using a P-97 micropipette puller (Sutter Instrument Company). Following injection, the oocytes were stored in Ringer’s solution (OR-2-Ca^2^-pen-strep) at 17–19 °C for 6–9 days on a rocker. This solution consists of (in mM unless noted): 82 NaCl, 2.5 KCl, 1 Na_2_HPO_4_, 5 HEPES, 1 CaCl_2_, 1 MgCl_2_, 0.5 theophylline, 100,000 units penicillin, and 10 mg streptomycin, set at a pH = 7.5. After 9 days, any oocytes not recorded on were stored at 4 °C to stop further maturation of the cell until the oocyte was used for recordings.

Recordings of the cell’s electrical activity were obtained with a two-electrode voltage clamp. Traces were recorded using Clampex 9.2 software and analyzed on ClampFit 9.2 (Molecular Devices). Various concentrations of acetylcholine (ACh) were suspended in OR-2 solution and were perfused (1 psi) over the oocyte. Recordings of the cell’s responses were recorded in response to the application of ACh at concentrations between 100 nM and 100 mM. Except for the desensitization tests, ACh was perfused for 3 s with an intervening wash of 90 s. To measure the degree of desensitization, the EC_85_ (effective concentration to elicit 85% of maximum response) for each subtype was applied for 60 s, with a 2 min wash between applications (performed in replicates of 3).

For voltage clamp recordings, the oocytes were clamped at −60 mV, while solutions were perfused at 17 mL∙min^−1^. The IV plot was generated by clamping the oocyte at −90, −75, −60, −45, −30, −15, and 0 (all in mV), and then we performed the same procedures as the voltage clamp recordings. All ligands were dissolved in OR-2-Ca^2+^ without theophylline, streptomycin, and penicillin. Solutions were perfused using an 8-valve (pinch), computer-controlled, pressurized perfusion system (Automate Scientific Inc.). Oocytes were impaled with borosilicate glass microelectrodes, filled with 3 M KCl and a resistance between 0.1 and 2 MΩ. Clampex 9.2 was used to run the electrophysiology protocols through a GeneClamp 500B amplifier and Digidata 1322A digitizer (Molecular Devices). Data were sampled at 5 kHz and filtered at 2 kHz. Peak amplitudes ranged from 3 nA to 200 nA. The range in peaks was dependent on each oocyte’s protein expression level, as well as the concentration of acetylcholine used to generate the response.

All recordings were normalized to the appropriate maximum response (E_MAX_) for each cell. Upon the collection of the recordings, we used Clampfit to analyze multiple parameters for each recording, including rise time, half-width (width of the peak at 50% of the peak amplitude), and desensitization. For our analysis, we compared the 1:5 and 5:1 results at the EC_50_ (10 µM, 333 µM, respectively) and the EC_85_ (333 µM, 10 mM, respectively, effective concentration 85% of maximum response). However, for desensitization we compared the EC_85_. The steady state currents measured at the 30 s mark (during a 60 s ACh application) were used for statistical analysis to compare the α3β2 receptor subtypes (see Section 4.1).

Controls were performed to ensure that neither the hα3 nor the hβ2 mRNA are able to form functional nAChRs by themselves. Following the previously outlined methods, we injected solely hα3 mRNA or hβ2 mRNA, waited 7 days, and then performed electrophysiological recordings with ACh. The recordings were under the same conditions as all other recordings. We found no evidence that the hα3 or the hβ2 forms a functional homomeric receptor.

### 4.1. Data and Statistical Analysis

Previously reported single-cell RT-qPCR data [6] were examined to determine if changing the subunit stoichiometry of the hα3 and the hβ2 nAChR subunits provided evidence for more than one receptor subtype. Microsoft Excel was used to analyze the data for all figures except Figure 2 and Figure 4. Specifically, the “=AVERAGE()” and “=STDEV()/(SQRT(COUNT()))” were used to calculate the mean and standard error of the mean. These values were then used to generate Figure 1 using “Insert Bar Graph” (Excel, 2013). Likewise, Figure 5a,b and Figure 6 were generated using the same calculations and tools. Also, the approximate EC_50_ and approximate EC_85_ were used as the points of comparison for the kinetic parameters (rise time and half-width). The data points for Figure 3 were also generated using the above stated formulas for the mean and standard error of the mean. However, a scatterplot fit with a linear trendline is more appropriate to present this data than a bar graph. Only the first 5 points of Figure 3 were fit for the trendline, because the data show a strong inwardly rectifying channel. Fitting the line linearly better estimates the reversal potential of an inwardly rectifying channel. Figure 4 was generated using GraphPad Prism v. 4. The data was fit using the “sigmoidal dose–response (variable slope)” tool. The hill slopes (n_H_), EC_50_ values, and R^2^ values, as well as their respective standard errors, were obtained from the curve fit analysis. Grubb’s outlier tests were used for all data sets (alpha = 0.05). For statistical tests, *p* < 0.05 is used as the level to determine significance (significant comparisons are marked with *). We report all means and standard errors of the mean as $\bar{x}$ ± SEM. GraphPad InStat v. 3 was used to calculate all *t*-tests and ANOVAs. Tukey post hoc tests were performed only if the *p* < 0.05. Figure 2a,b are example traces that were collected using Clampex 9.2 and analyzed with Clampfit 9.2 (Axon Instruments).

### 4.2. Materials

Plasmids: hα3 (Origene# SC126406) and hβ2 (Origene# SC309051), both in the pCMV6-XL4 plasmid, were purchased from OriGene Technologies Inc., 9620 Medical Center Drive, Suite 200, Rockville, MD, USA, 20850.

One Shot^®^ *E. coli* chemically competent cells (Invitrogen, Waltham, MA, USA) were transfected with the plasmids and used to grow the plasmids. Thermo-Fischer Scientific 168 Third Avenue, Waltham, MA, USA, 02451.

The HiSpeed^®^ plasmid purification kit was used to isolate and purify the plasmids from the *E. coli*: Qiagen, 19300 Germantown Road, Germantown, MD, USA, 20874.

The mMessage mMachine^®^ T7 Ultra Kit was used to generate the mRNA from the plasmids: Thermo-Fischer Scientific 168 Third Avenue, Waltham, MA, USA, 02451.

SacI restriction enzyme was used to linearize the plasmids prior to mRNA generation: New England Biolabs, 240 Country Road, Ipswich, MD, USA, 01938-2723.

Tris-EDTA Buffer: BioExpress Corporation, 420 N Kays Dr, Kaysville, UT, USA 84037.

Defolliculated *Xenopus laevis* oocytes were obtained from Ecocyte BioScience, 111 Ramble Ln #109, Austin, TX, USA, 78745.

Oocyte injection, Nanoject II microinjector: Drummond Scientific Company, 500 East Park Way, Broomall, PA, USA, 19008.

Injection (1.12 mm OD × 0.51 mm ID) and recording (1.5 mm OD × 1.17 mm ID) capillary glass was obtained from: Harvard Apparatus, 84 October Hill Road, Holliston, MA, USA, 01746.

Injection pipettes and recording electrodes were pulled using Model P-97 puller: Sutter Instrument Company, 1 Digital Drive, Novato, CA, USA, 94949.

Data acquisition software for electrophysiological recordings (Clampex 9.2) and recording analysis (Clampfit 9.2). GeneClamp 500B amplifier. Digidata 1322A digitizer. Axon Instruments, Molecular Devices, 1311 Orleans Drive, Sunnyvale, CA, USA, 94089.

Reagents for OR-2 Ca^2+^ solution: NaCl, KCl, Na_2_HPO_4_, HEPES, CaCl_2_, MgCl_2_, theophylline, penicillin, streptomycin; Acetylcholine chloride: Sigma-Aldrich, 3050 Spruce Street, St. Louis, MO, USA, 63103.

Pinch valve perfusion system (product #s 13-pp-54, and 09-08) was purchased from Automate Scientific Inc., 3271 Adeline Street, Unit B, Berkeley, CA, USA, 94703.

GraphPad Prism v. 4 and GraphPad Instat v. 3.05: GraphPad Software Inc., 7825 Fay Ave #230, La Jolla, CA 92037 USA.

Microsoft Office Excel 2013: Microsoft Building 92, 15010 NE 36th St, WA 98052-6399 USA.

## Figures and Tables

**Table 1:** Summary of ACh Dose–Response Curves.

	1:5 α3:β2 mRNA	5:1 α3:β2 mRNA
EC-50 (µM ACh) ^1^	12 ± 1.7	264 ± 1.6
Hill Slope	0.48 ± 0.13	0.55 ± 0.15
R^2^	0.74	0.77

^1^ comparison of acetylcholine EC-50 values with ANOVA shows statistically significant difference, *p* < 0.0001.

## Data Availability

The original contributions presented in this study are included in the article. Further inquiries can be directed to the corresponding author.

## Abbreviations

The following abbreviations are used in this manuscript:

ACh	Acetylcholine
AD	Alzheimer’s Disease
ADHD	Attention Deficit Hyperactivity Disorder
ASD	Autism Spectrum Disorder
CA1	Amun’s horn
CNS	Central nervous system
n_H_	Hill slope
nAChR	Nicotinic acetylcholine receptor
PNS	Peripheral nervous system
RT-qPCR	reverse transcription-quantitative polymerase chain reaction
